# Topological optimization and fatigue life prediction of a single pad externally adjustable fluid film bearing

**DOI:** 10.1038/s41598-024-64259-2

**Published:** 2024-06-10

**Authors:** Harishkumar Kamat, Anand Pai, Navaneeth Krishna Vernekar, Chandrakant R. Kini, Satish B. Shenoy

**Affiliations:** 1 Department of Mechanical and Industrial Engineering, Manipal Institute of Technology, Manipal Academy of Higher Education, Manipal, Karnataka 576104 India; 2 Department of Aeronautical and Automobile Engineering, Manipal Institute of Technology, Manipal Academy of Higher Education, Manipal, Karnataka 576104 India

**Keywords:** Single pad, CFD, Fatigue, Life prediction, FSI, Topology optimization, Mechanical engineering, Aerospace engineering

## Abstract

This work focuses on the prediction and comparison of the fatigue life of topologically optimized pads in an externally adjustable fluid film (EAFF) bearing. It integrates one-way/two-way fluid–structure interaction analysis, topological optimization (TO), and design modifications of the pad of an externally adjustable fluid film bearing. The major goal is to create an optimum pad design that minimizes weight and maintains structural integrity, and then to predict and compare the fatigue life of these alternative designs. The outcomes of the present study are as follows: (i) Two-way FSI results show a decrease of 65.64% in hydrodynamic fluid film pressure when compared to one-way FSI results because they take into account modifications in the fluid region's geometry caused by pad deformation; (ii) even though the maximum pad deformation in optimized pad geometry (Type-4) resulting from oil film pressure is relatively small (0.0036551 mm), the influence of pad deformation on the fluid domain due to hydrodynamic fluid film pressure cannot be understated; and (iii) when comparing the TO technique's results with fatigue life results, four elongated holes in the radial direction (Type-4) are most appropriate.

## Introduction

Fatigue has been a subject of study for over 150 years. Understanding the fatigue behavior of materials is crucial for designing reliable and durable structures and components. Continuous application of cyclic loads leads to material failure after a specific number of loading/unloading cycles. The failure may occur even if the cyclic stress level is much lower than the material's ultimate stress or yield point^1–3^. Most structural failures are attributed to fatigue and often occur without warning^3^. Fatigue failure is linked to plastic deformation. The behavior of materials in critical locations, such as notches, is influenced by strain.

Stress life (SN) and strain life (EN) are two fundamental approaches used in fatigue analysis to predict the fatigue life of materials. The stress life approach is particularly useful when the material behaves elastically, and its performance is primarily influenced by stress rather than plastic deformation. However, components with notches, welds, or other structural irregularities may result in plastic deformation^2^. Hence, under these conditions, the strain life (EN) approach is used to predict the fatigue life of a component more effectively. The EN approach is also used when the load history is random or irregular^1^. Various factors, such as cyclic stress state, geometry, surface quality, material type, residual stresses, load direction, temperature, distribution of internal defects, and their size, affect the fatigue life of a machine component under cyclic or random loading. The fatigue life of a machine component can be altered by controlling these influencing parameters.

The study of journal bearings has been a subject of significant interest over the past five decades. Numerous theoretical, experimental, and computational methods have been used to study the performance of journal bearings and their profiles. Advances in computer technology and the application of Computational Fluid Dynamics (CFD), as well as Fluid–Structure Interaction (FSI) methodologies, have been beneficial in studying the performance of journal bearings. Chen and Hahn^4^ demonstrated the use of CFD software packages for steady-state hydrodynamic cases for various bearings. Gertzos et al.^5^ employed and validated^6,7^ a CFD model to examine the performance characteristics of a journal bearing lubricated with a Bingham fluid across various L/D ratios. Bompos and Nikolakopoulos^8^ conducted CFD analysis on a journal bearing lubricated with magnetorheological fluids and having different L/D ratios. Yiping et al.^9^ and Shenoy et al.^10^ studied the hydrodynamic behavior of a 360° journal bearing using ANSYS FLOTRAN software. Shenoy et al.^10^ performed CFD analysis to determine fluid pressure values in the laminar zone with different L/D ratios and eccentricity ratios (ε). They subsequently conducted structural analysis to determine bearing liner stresses and deformation. Manshoor et al.^11^ examined various turbulence models in a three-dimensional CFD investigation on an oil-lubricated journal bearing, analyzing the influence of each turbulence model on bearing performance. Chen et al.^12^ used CFD-coupled FSI analysis to examine high-speed applications of journal bearing surfaces. The study focused on investigating how the number and location of oil grooves affect the bearing surface. In continuation, Lin et al.^13^ employed the FSI technique to examine the impact of the number of oil groove positions on the bearing surface. Based on these important CFD/FSI related literatures it is found that reduction in fluid film pressure value was observed by considering the elastohydrodynamic effect. Hence, the impact of elastic deformation on the bearing performance cannot be neglected^14–18^. Although oil film pressure-induced bearing deformation is relatively small, its importance cannot be understated. In this study, topology optimization (TO) is also employed to make the pad of an EAFF bearing lightweight by maintaining structural integrity. The most prominent topology optimization methods include the Solid Isotropic Material with Penalization (SIMP) technique^19^, the Evolutionary Structural Optimization (ESO) technique^20^, and the level-set method^21,22^. SIMP, the most prevalent technique, predicts optimal material distribution within a designated design domain. Because of its ease of conceptualization and numerical execution, the SIMP model has emerged as the most popular and effective topology optimization tool^19,23^.

Fatigue life prediction enables the appropriate maintenance of equipment and provides insights into the decline in performance over time. Cyclic loading in the bearing may be due to rotor imbalance or fluctuations of fluid dynamic forces, which may cause mechanical damage. An unstable vibration of a pad that causes it to oscillate back and forth repeatedly is called pad fluttering. This condition may cause wear or damage to the tilting pad bearings^24^. Thomas^25^ reported fatigue failure due to pad fluttering in the tilting pad bearing. Yang et al.^24^ developed a modified tilted pad bearing model and experimentally proved the reduction of pad fluttering. The model includes a groove and wedge shape at the lead edge of the pad. Also, it was proved that the modified tilted pad bearing model develops adequate dynamic pressure in no-load conditions. Dong et al.^26^ predicted the fatigue life of a tin-based Babbitt journal bearing bush using an experimental and numerical technique. After performing an initial Fluid–Structure Interaction (FSI) study at several eccentricity ratios, the fatigue life of the bearing bush was assessed using nCode 2023.1 DesignLife software^27^. Ding et al.^28^ and El-daher et al.^29^ investigated the remaining useful life of the journal bearing using both numerical and experimental work on multilayered journal bearing considering multiaxial stresses (both normal and shear stresses). They used modified Dang Van's criteria to numerically predict the life of the journal. Similarly, Sous et al.^30^ conducted experiments and finite element simulation to predict the safe and unsafe working conditions considering the multiaxial stress state of journal bearing coated with white metal. It was concluded that fatigue cracks are observed because of material overload and not because of asperities. Harris et al.^31^ investigated the fatigue life of roller bearing and compared the results with different bearing steel materials. Results indicated that hardened bearing steel M50 has a limitation of operating speed and load induced stresses. Meanwhile, with M50 Nil and M50 Super Nil as bearing material, operating speed increases, and fracture near the ring is eliminated completely. Qian et al.^32^ experimentally studied and presented a unique method to evaluate the remaining useful life and track the defects of ball bearing by a multi-time scale approach. Also, Harris and McCool^31^ studied the accuracy level of Lundberg–Palmgren (LP) and Ioannides–Harris (IH) methods, which are used for fatigue life prediction of roller bearings. Life calculated by experiments, LP, and IH methods are compared, and results showed that the IH method is superior in predicting the bearing life. It is possible to prevent bearing catastrophic disasters by forecasting the bearing's failure or remaining useful life. The research of fatigue life prediction is significantly impacted by advancements in computer performance and technical innovations such as artificial intelligence and machine learning techniques^33–35^. Therefore, it is essential to find the fatigue life through numerical study.

This paper uses fatigue and damage tolerance methodologies to predict the life of an EAFF bearing pad. The investigation is divided into three phases. Initially, a one-way/two-way Fluid Structure Interaction (FSI) is conducted with an EAFF bearing operating under varying conditions. In the second phase, based on the results of the FSI, the pad is then subjected to density-based Topology Optimization (TO). Subsequently, alternative pocket designs, such as circular and elongated holes, are introduced to the Topologically Optimized pads to reduce weight without sacrificing their structural integrity. In the final phase, based on the SN approach, the fatigue life of the modified pads is predicted and compared using nCode designLife. “Methodology and model descriptions” outlines the methodology utilized in the numerical investigation to simulate the interaction between fluid and structure in one-way and two-way FSI studies employing the ANSYS Workbench 2022 R2 numerical tool. This chapter also covers descriptions of the models employed in the present numerical analysis. “Mesh sensitivity analysis” highlights the grid generation and a mesh sensitivity analysis adopted in the present study. “Results and discussion” focuses on the outcomes of one-way and two-way FSI analysis, the TO technique applied, and the life prediction of a pad of an EAFF bearing working under high rotor speed applications. Finally, “Conclusion” presents an overview of the findings.

## Methodology and model descriptions

### Methods

**Figure 1:**
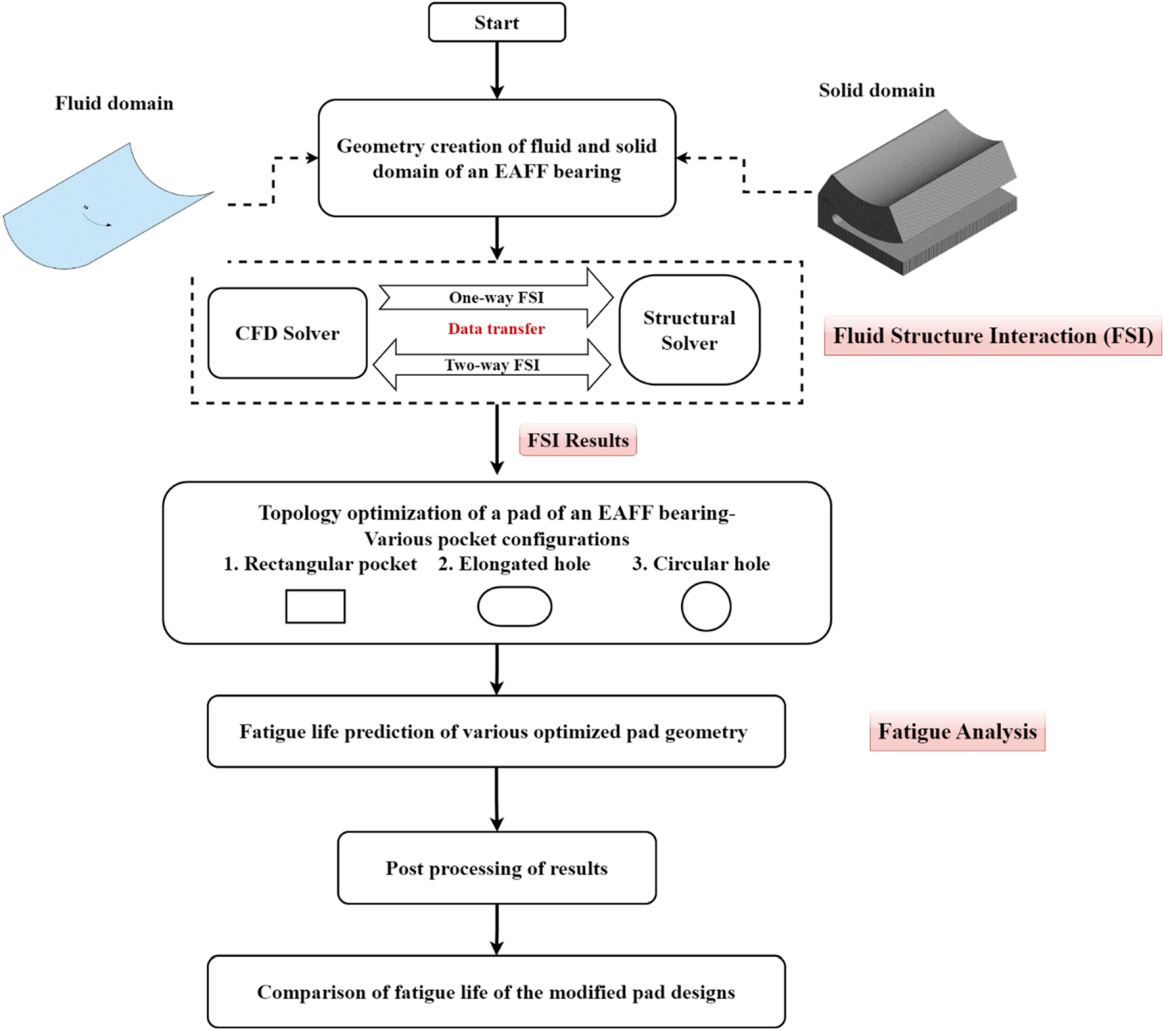
Flowchart illustrating the steps employed for fatigue analysis.

The flowchart shown in Fig. 1 explains the various stages of the present study and the procedures adopted for life prediction of an EAFF bearing pad. Initially, a base model (Type-0) of a single pad EAFF bearing, including both fluid and solid domains are created using CAD tools. Subsequently, the FSI method is employed to analyze the performance of a pad of an EAFF bearing. Later, using the results of a two-way FSI, the TO technique is employed to minimize the bearing's weight by implementing a range of pocket configurations, including circular and elongated holes. Finally, nCode DesignLife numerical tool is used for fatigue analysis for different pocket configurations.

### Computational model and boundary conditions

**Figure 2:**
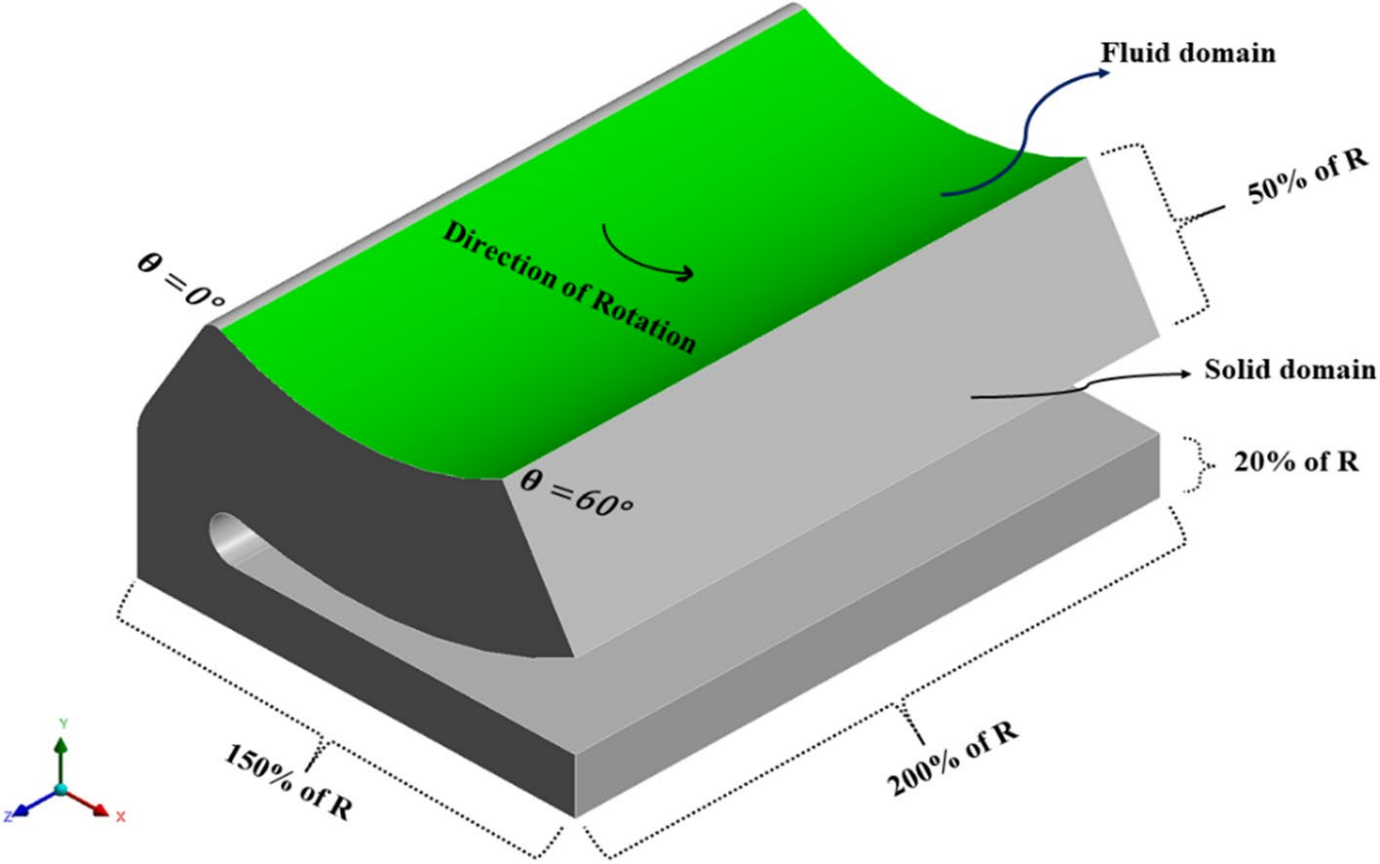
Pictorial representation of EAFF bearing with fluid and solid domain with parametric dimensions in terms of journal radius (R).

**Figure 3:**
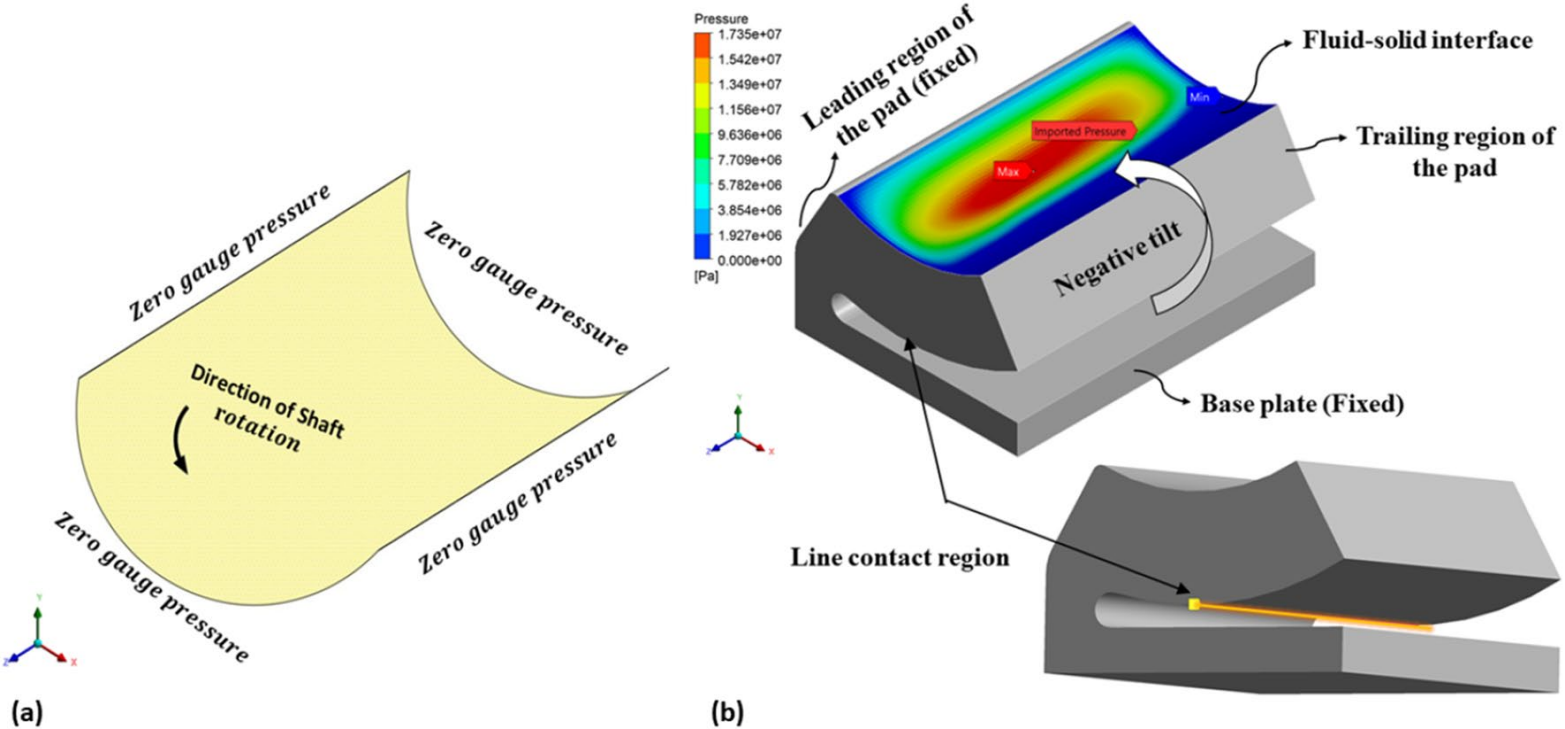
Boundary conditions used in the present study (**a**) fluid region and (**b**) solid region.

**Figure 4:**
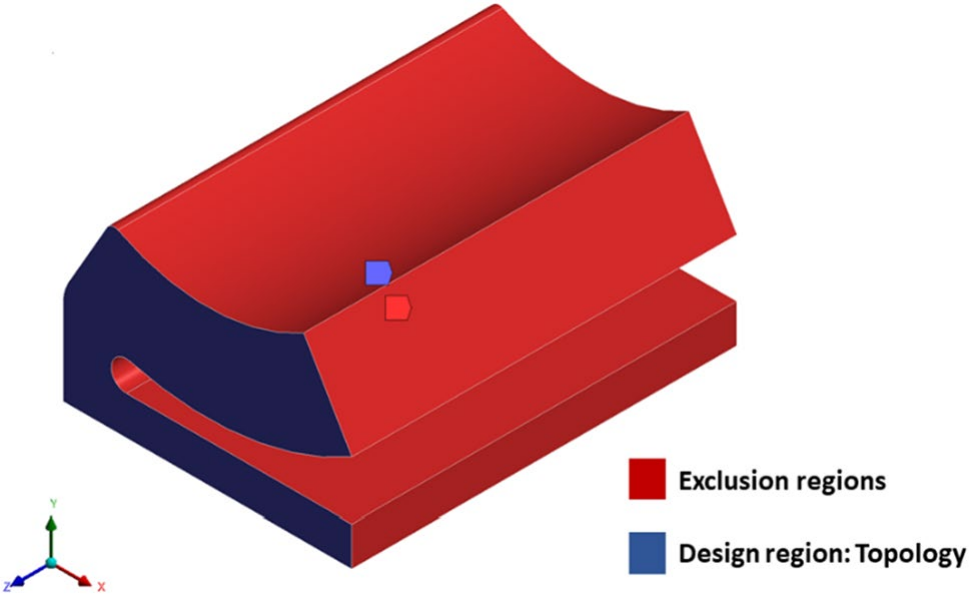
Pictorial representation of design region and exclusion region.

**Figure 5:**
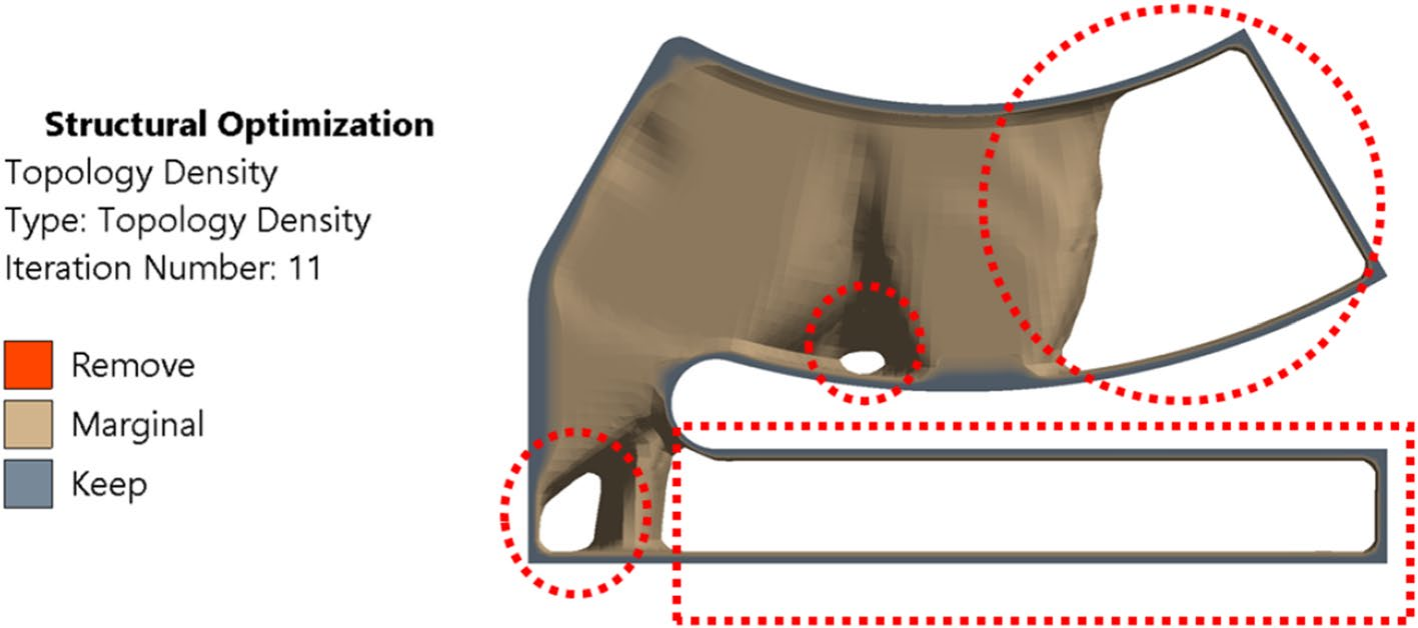
Recommendations of TO technique for optimum material from pad geometry.

**Figure 6:**
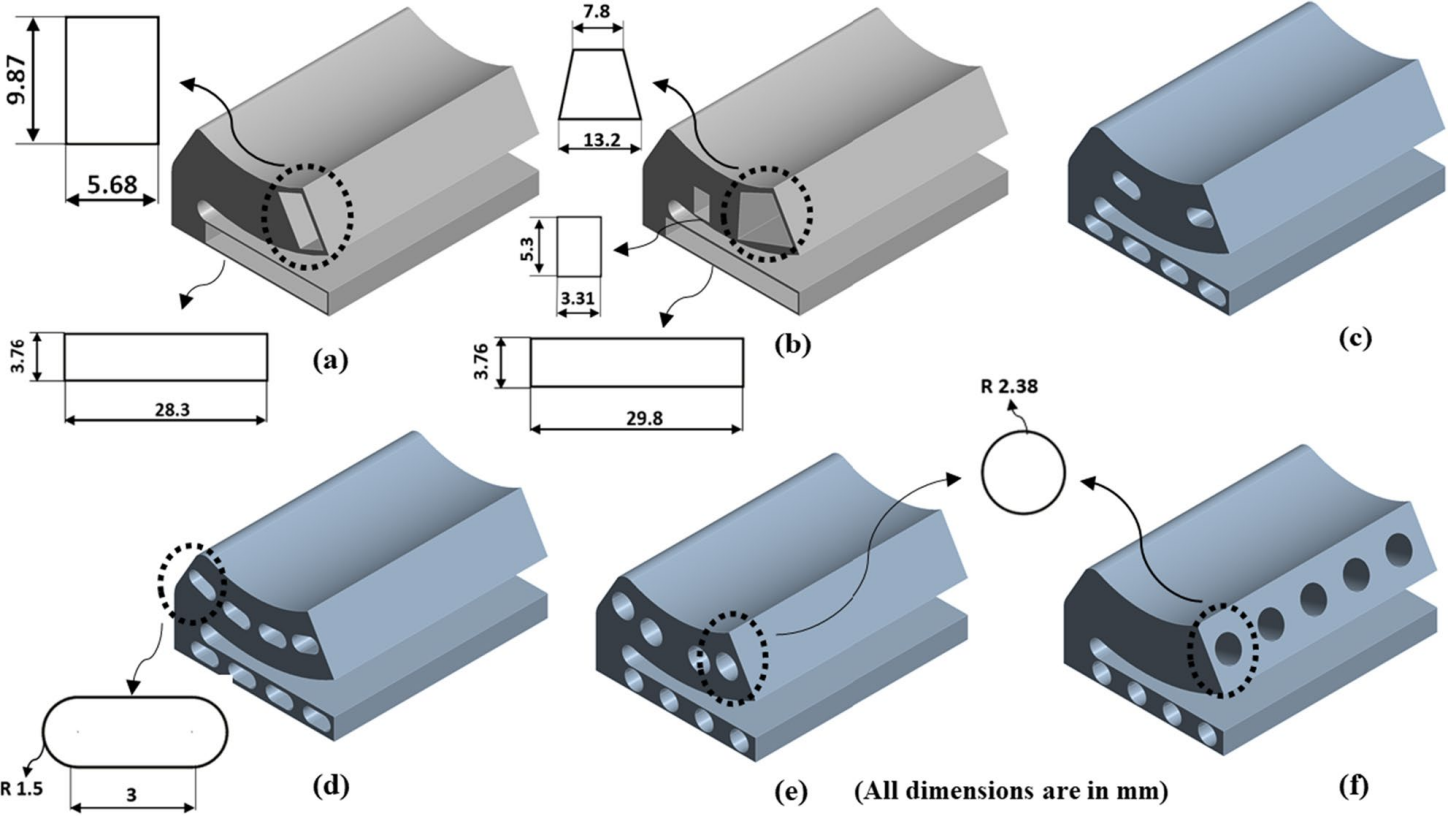
Various pocket configurations adopted in the present study (**a**) Type-1 and (**b**) Type-2 (as per the recommendation of TO technique), (**c**) Type-3, (**d**) Type-4, (**e**) Type-5, and (**f**) Type 6.

**Figure 7:**
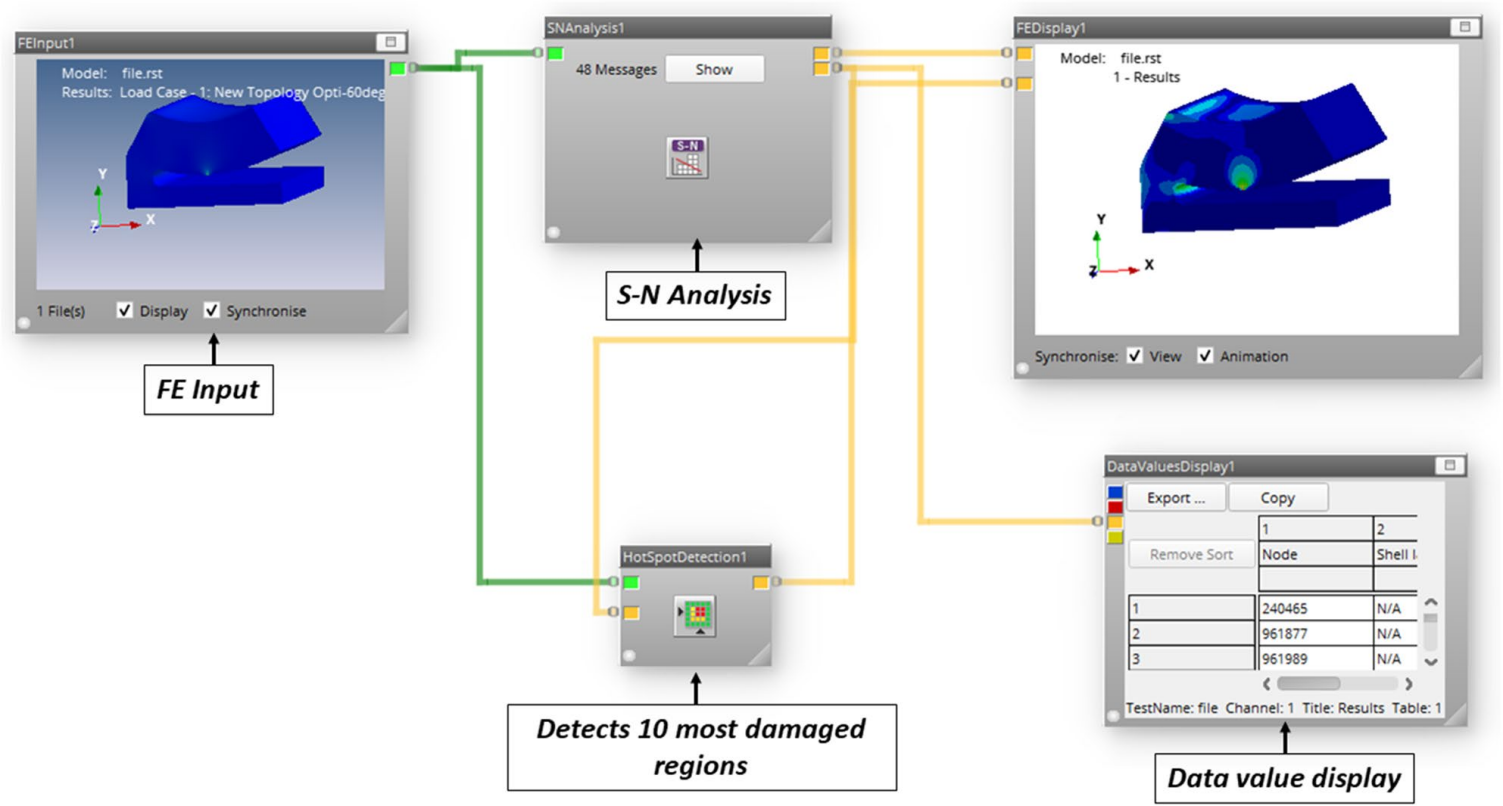
Basic architecture of the stress life (SN) method used in the nCode DesignLife software.

The shape of the base model, as in Fig. 2 is inspired by the adjustable bearing configuration patented by Parkins and Martin^36^. Various dimensions of the base models are parametrized in terms of journal radius. Later, FSI analysis using ANSYS Fluent is carried out to study the one-way/two-way interaction between fluid and structure within the single pad in an externally adjustable fluid film bearing. Figure 3 provides a pictorial representation of the boundary conditions used for the FSI analysis. One-way FSI considers the effect of fluid on the pad structure, while two-way FSI involves a mutual interaction where the pad structure affects the fluid and vice versa. Based on the findings from the FSI analysis, topological optimization (TO) of the bearing pad is performed. Topological optimization aims to enhance the structure's performance by redistributing material, usually to reduce weight while maintaining or improving structural integrity. The goal is to achieve an optimized design that meets structural requirements while minimizing weight. Faces of pad geometries like fluid–solid interface, base plate, trailing, and leading region are excluded from the TO technique as these faces need to be retained from the design point of view, as shown in Fig. 4. The output of TO as shown in Fig. 5, provides optimum material layout for the pad by removing materials from the low deformation and stress region^37,38^. Red circles indicate the region of the material to be removed, and gray indicates the region where the material is retained. This optimized material distribution is a key input for the subsequent modifications and pocket designs in the pads. Utilizing the material layout derived from topological optimization, specific modifications, and pockets are created in the pad (Type-1 to Type-6), as shown in Fig. 6. The final step involves predicting and comparing the fatigue life of the modified pad designs based on the SN approach. This is done using nCode DesignLife software, as in Fig. 7.

**Figure 8:**
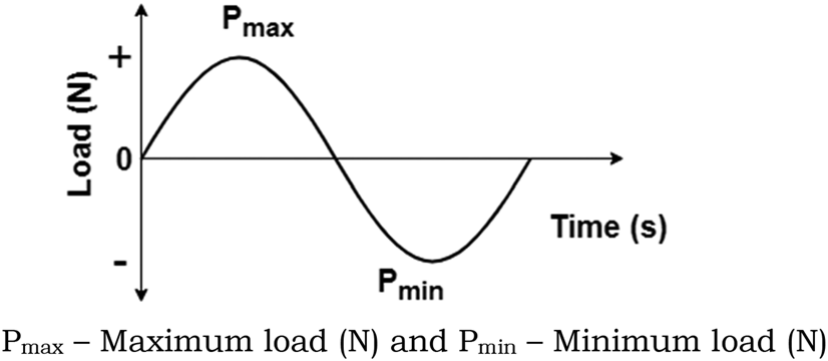
Pictorial representation of fully reversed loading (R_r_ = − 1).

Table 1 specifies the various operating parameters considered in this study and S–N data of material is included in the Supplementary Material file (Supplementary Fig. S1). Fatigue analysis is conducted on both non-optimized (Type-0) and optimized (Type-1 to Type-6) designs. During the preprocessing stage, Finite Element (FE) results are imported, and parameters for loading type and material properties are defined. The loading type is characterized by constant amplitude fully reversed loading, with a load ratio (Rr = Pmin/Pmax) set at − 1, as presented in Fig. 8. In fatigue analysis, the cyclic load acting on the pad geometry is derived from fluid film pressure generated due to hydrodynamic action during bearing operation. This cyclic load, originating due to fluid pressure, acts as a primary factor for initiating cracks within the pad geometry, posing a potential risk of fatigue failure in the future.

**Table 1:** Dimensional specification adopted in the current study^39^.

Specifications	
Radius of journal (R)	23.8 mm
Pad angle	60°
Radial clearance (C)	48 μm
Pad material^40^	Bronze material
Length to diameter ratio (L/D)	1.0
Shaft or journal speed (N)	7500 rpm
Eccentricity ratio (ε)	0.6
Pad tilt adjustments	− 0.0061° at leading edge

Lubricant properties	
Viscosity (η)	1.25e−2 Ns/m^2^
Density	840 kg/m^3^

## Mesh sensitivity analysis

**Figure 9:**
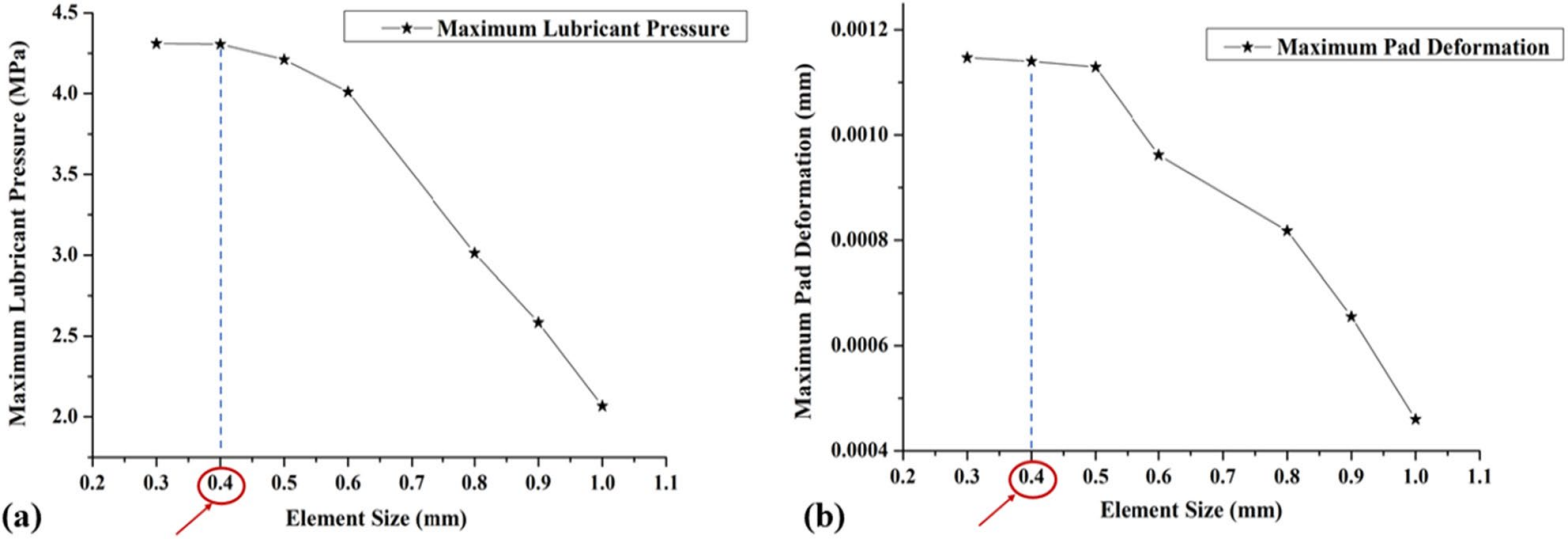
Comparison of (**a**) maximum fluid pressure and (**b**) maximum pad deformation for various element size.

Mesh sensitivity analysis is essential for identifying the appropriate mesh resolution required to obtain accurate numerical simulations. Mesh sensitivity analysis reduces computational expenses and preserves result accuracy in finite element analysis and computational fluid dynamics simulations by fine-tuning the element size. In the present study, a mesh sensitivity analysis is carried out on the pad geometry and fluid domain by changing the element sizes from 1 to 0.3 mm. The results of this analysis, as illustrated in Fig. 9, reveal that the variation in Fluid–Structure Interaction (FSI) results between element sizes of 0.4 mm and 0.3 mm is less than 2%. As a result, an element size of 0.4 mm is suitable for further investigations, as the small variation indicates that the choice of element size within this range does not significantly impact the results.

## Results and discussion

The current investigation is based on the result that the bearing works better when the pad is modified in the upward direction (negative), which was reported in the earlier research study^41–43^. In negative pad adjustments, clearance space between the rotor and the pad geometry is reduced, which hence increases hydrodynamic pressure generation. These pressure values are also maximum when the eccentricity ratio is higher. Thus, fatigue analysis is performed using the numerical findings of FSI and topology optimization as explained in the flowchart shown in Fig. 1.

### Results of fluid structure interaction study

In a one-way FSI analysis, a computational fluid dynamics (CFD) analysis is performed first, followed by a structural analysis. In two-way FSI analysis, results of fluid pressure, deformation, and stress exchange between fluid and structural domains. This fluid pressure acts like an external force on the pad bearing, leading to structural deformation.

**Figure 10:**
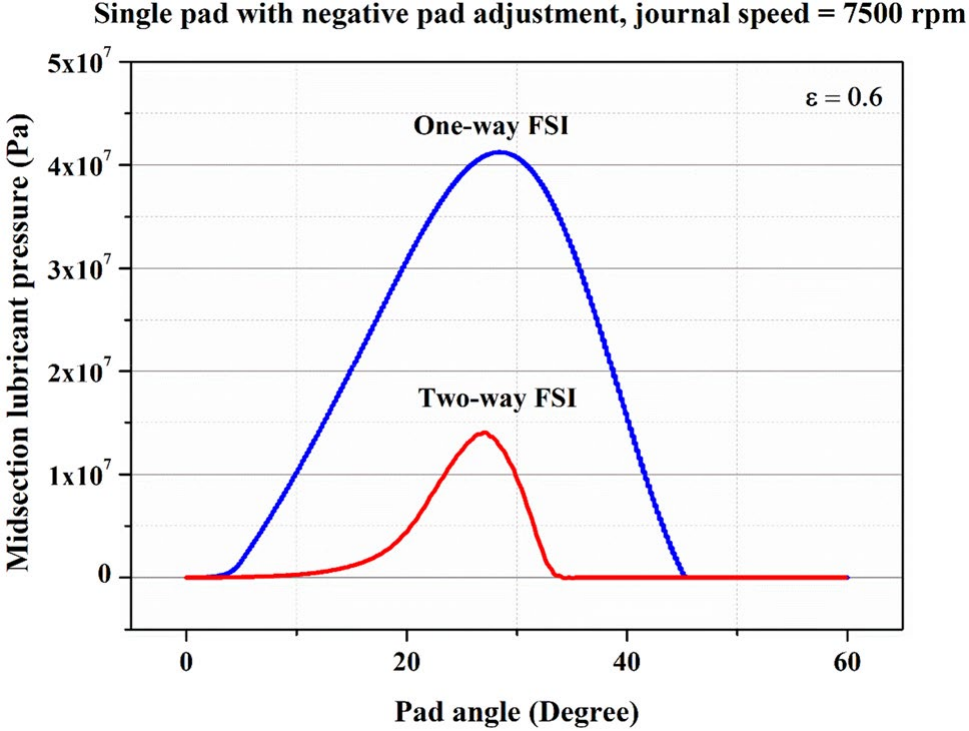
Fluid pressure distribution in the bearing in the midsection for one-way and two-way FSI at 7500 rpm journal speed.

Figure 10 displays a comparison of fluid pressure results taken from the midsection for both one-way and two-FSI. Notably, the highest fluid pressure obtained in the two-way FSI is 1.417e7 Pa, where fluid pressure is reduced by 66% in two-way FSI compared to one-way. A similar trend is seen for pad deformations and stresses. In two FSI, due to the utilization of the ANSYS dynamic meshing technique, the system effectively captures alterations in the fluid domain induced by pad deformation. This leads to reduced fluid pressure and stresses compared to the results obtained through one-way FSI.

**Figure 11:**
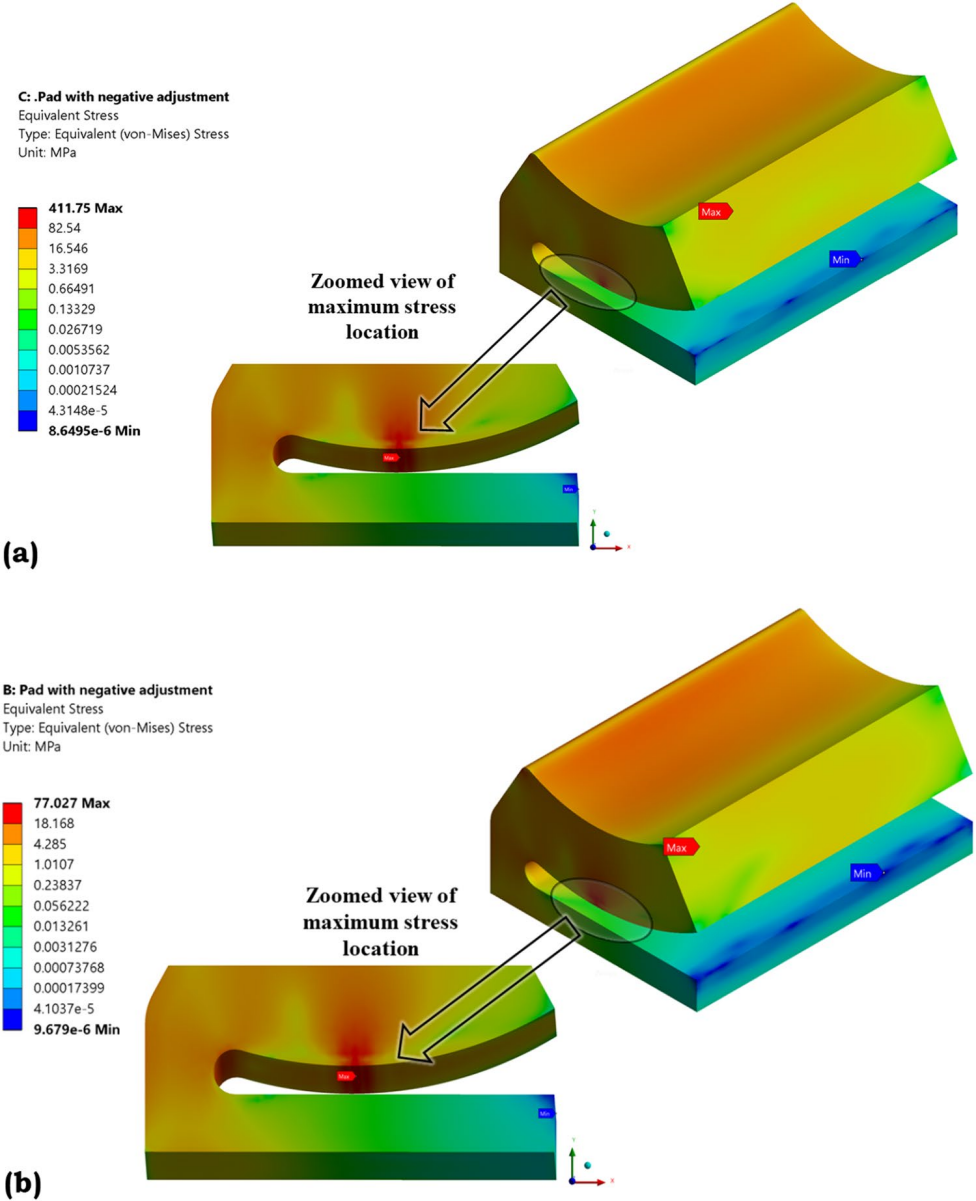
Location of maximum stresses in the pad (**a**) one-way FSI and (**b**) two-way FSI.

Based on FSI results it is also noted that high stresses are concentrated at the line contact region of a pad as shown in Fig. 11. These are due to the application of a special cam profile at the center of the pad for tilt adjustment and is a point of concern. Thus, using the 'nCode DesignLife' numerical tool, the fatigue life of a pad working under high-speed applications is predicted. Additionally, based on the topology optimization technique findings, the pad's life is predicted for various pocket configurations.

### Results of fatigue life prediction

The pad of an EAFF bearing is subjected to the density-based topology optimization (TO) method using the findings from the two-way FSI analysis. Based on the TO technique results, it is suggested that the mass of the pad geometry can be reduced by 42.91% (139.8 g) compared to the original geometry (245.06 g). The recommended pad geometry as per the TO method is shown in Fig. 5. Following the suggestions and findings, the pad geometry undergoes a redesign process that involves eliminating material from low-stress areas near the base plate and trailing edge by using pockets like elongated and circular holes.

**Figure 12:**
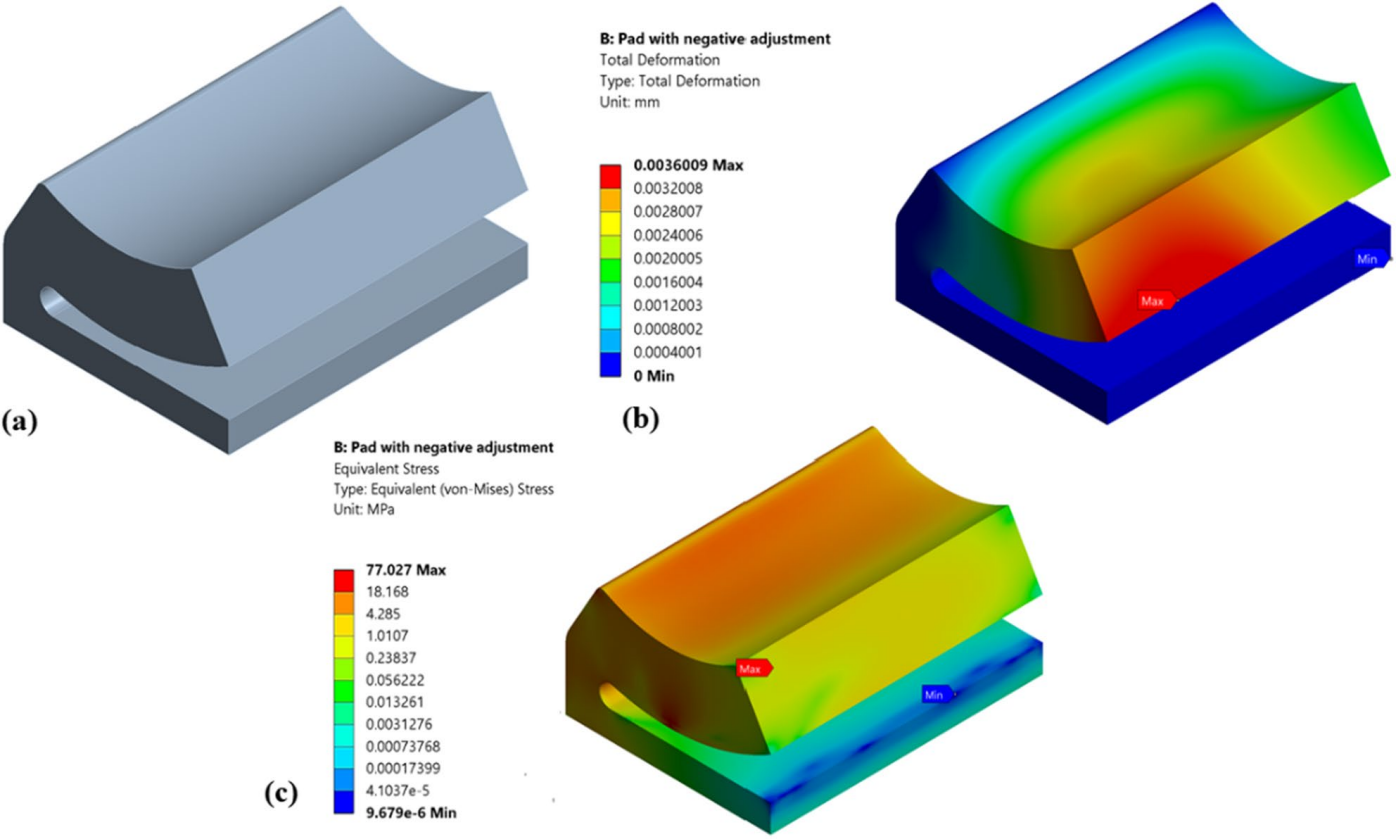
Two-way FSI results of non-optimized single pad EAFF bearing (Type-0) at 7500 rpm journal-speed (**a**) pad bearing, (**b**) pad deformation and (**c**) von-Mises stress.

**Figure 13:**
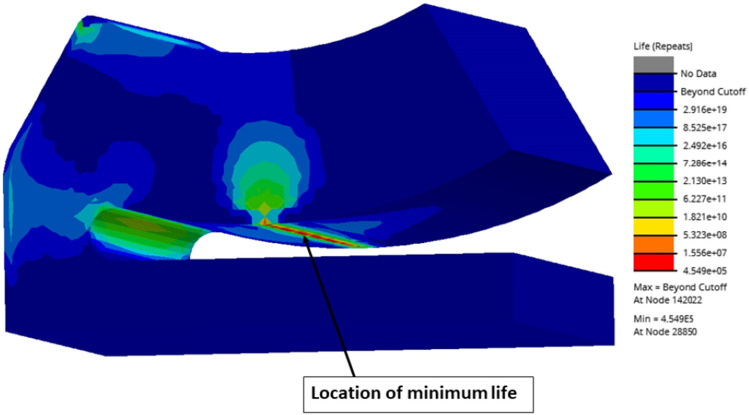
Fatigue life of non-optimized single pad EAFF bearing (Type-0) at 7500 rpm journal speed.

Initially, the structural and fatigue analysis results of various pocket configurations are compared with the results of non-optimized single pad EAFF bearing shown in Figs. 12 and 13. The fatigue results of the pad presented in Fig. 13 highlight the region of minimum life (4.549 e5 cycles), which is also the location of the maximum stresses predicted during FSI analysis. It can be seen that fatigue life is shorter in the region with larger stresses, and this area is more prone to wear and fatigue crack initiation. Although the pad is not experiencing fatigue failure under the current loading circumstances, the fatigue analysis results show a critical region.

**Figure 14:**
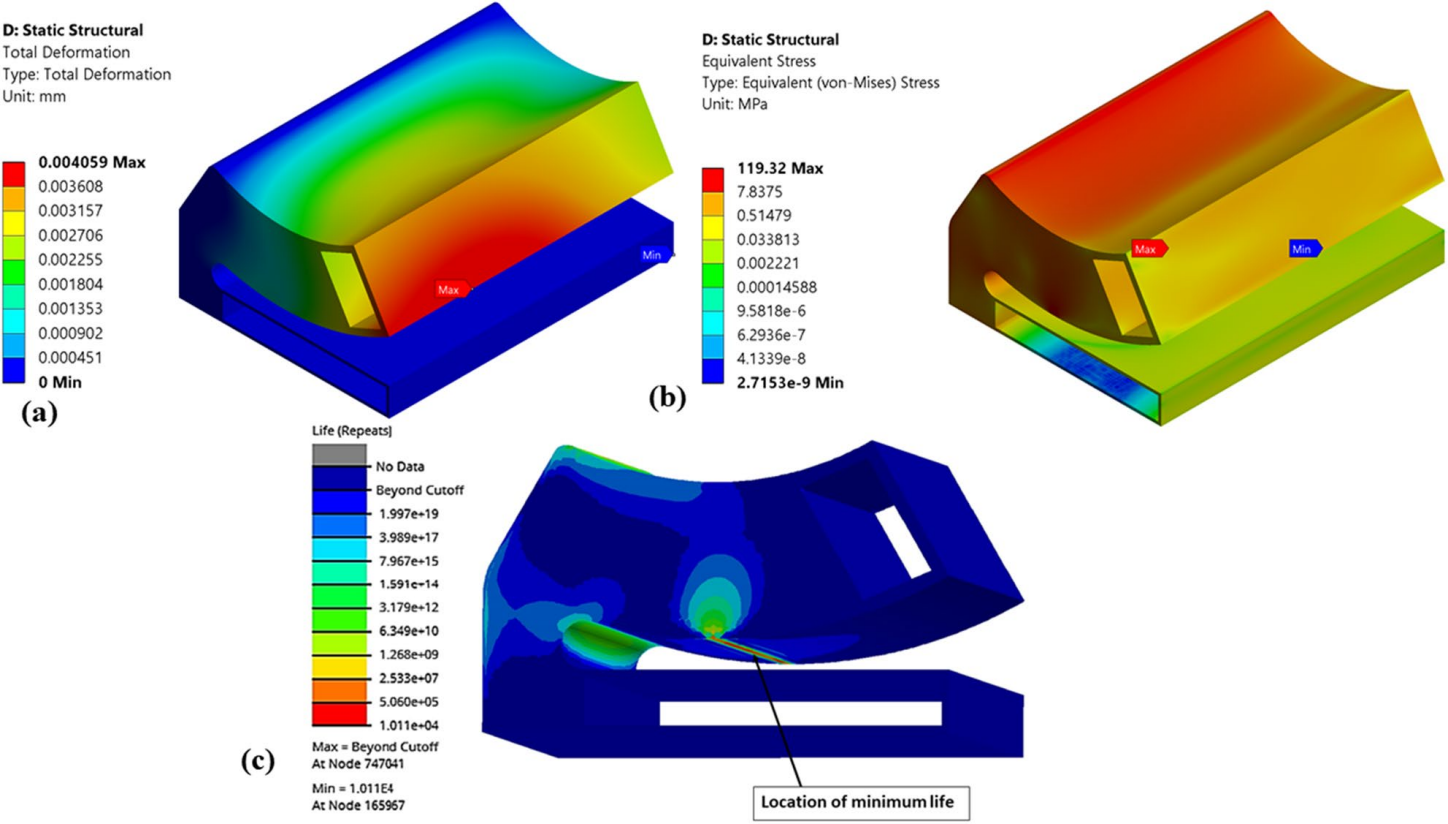
Numerical results of optimized (Type-1) single pad EAFF bearing (**a**) pad deformation, (**b**) von-Mises stress, and (**c**) fatigue life.

**Figure 15:**
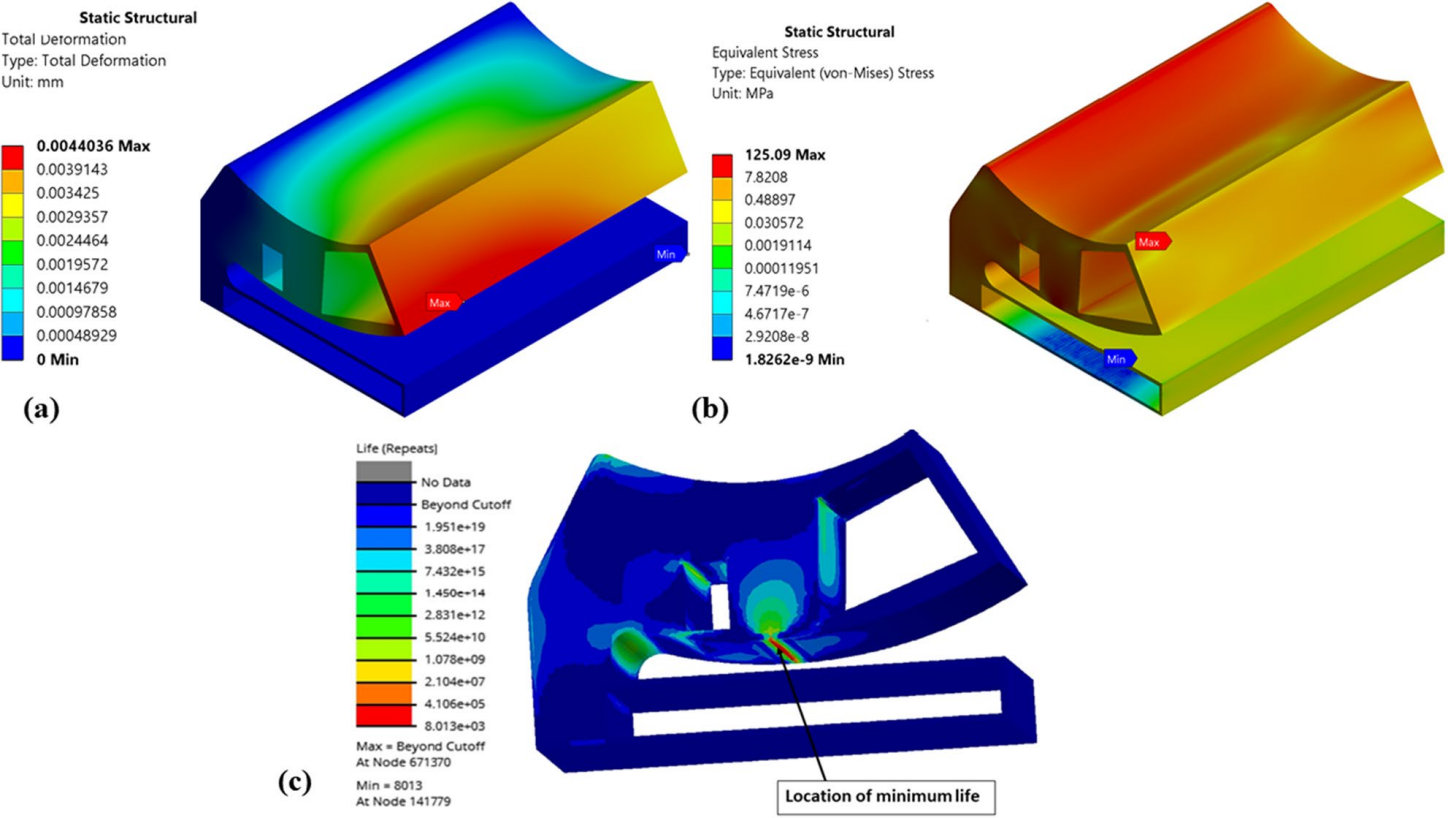
Numerical results of optimized (Type-2) single pad EAFF bearing (**a**) pad deformation, (**b**) von-Mises stress, and (**c**) fatigue life.

**Figure 16:**
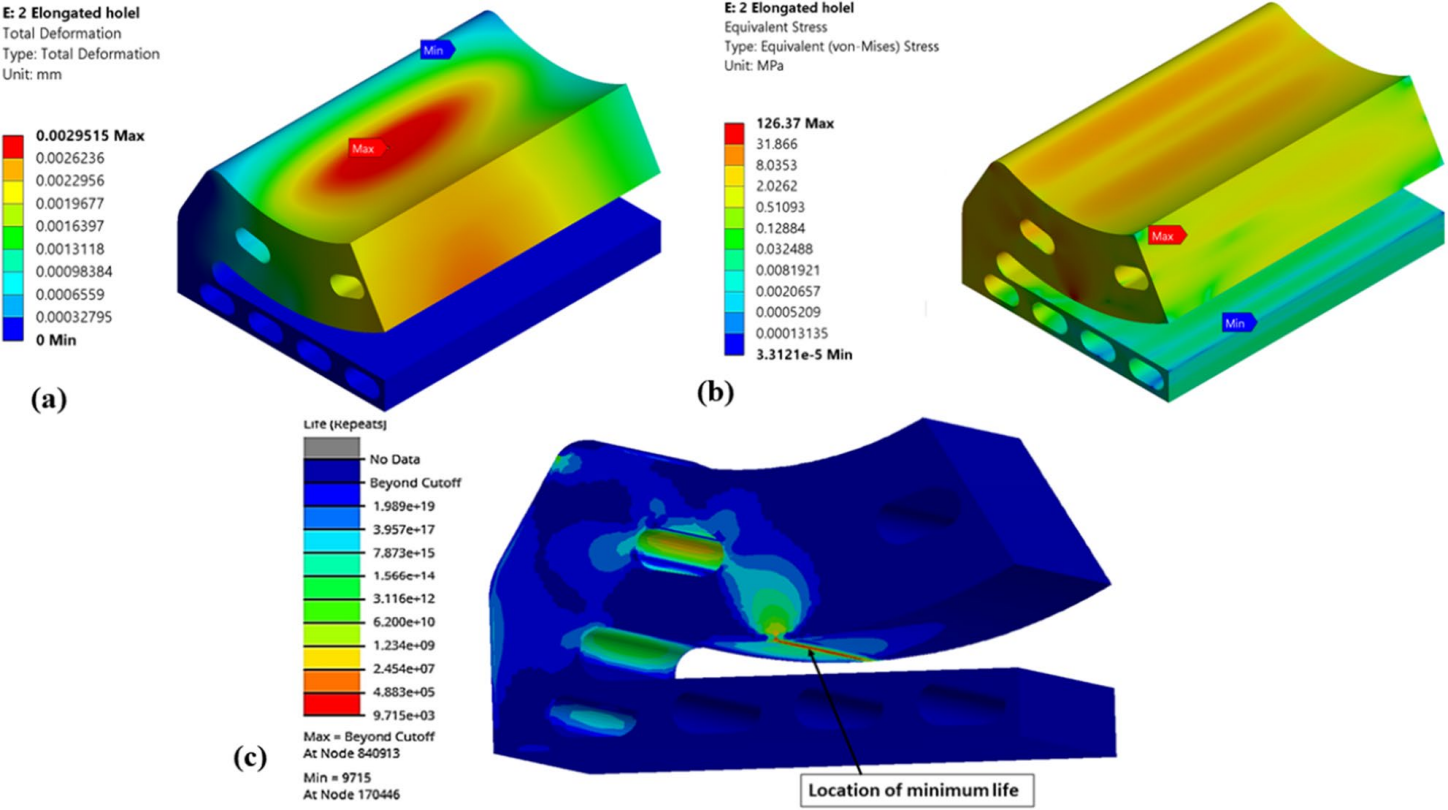
Numerical results of optimized (Type-3) single pad EAFF bearing (**a**) pad deformation, (**b**) von-Mises stress, and (**c**) fatigue life.

**Figure 17:**
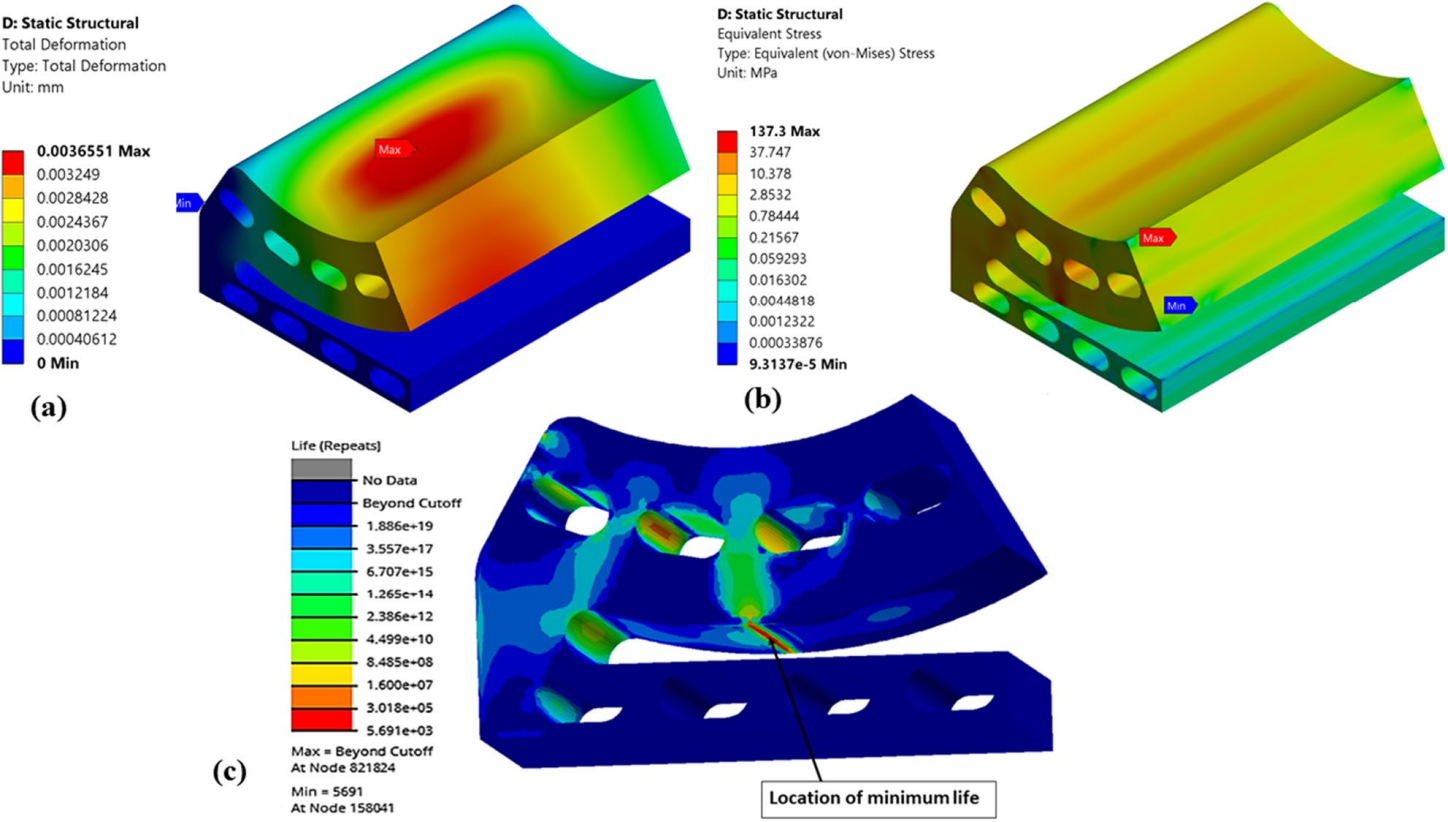
Numerical results of optimized (Type-4) single pad EAFF bearing (**a**) pad deformation, (**b**) von-Mises stress, and (**c**) fatigue life.

**Figure 18:**
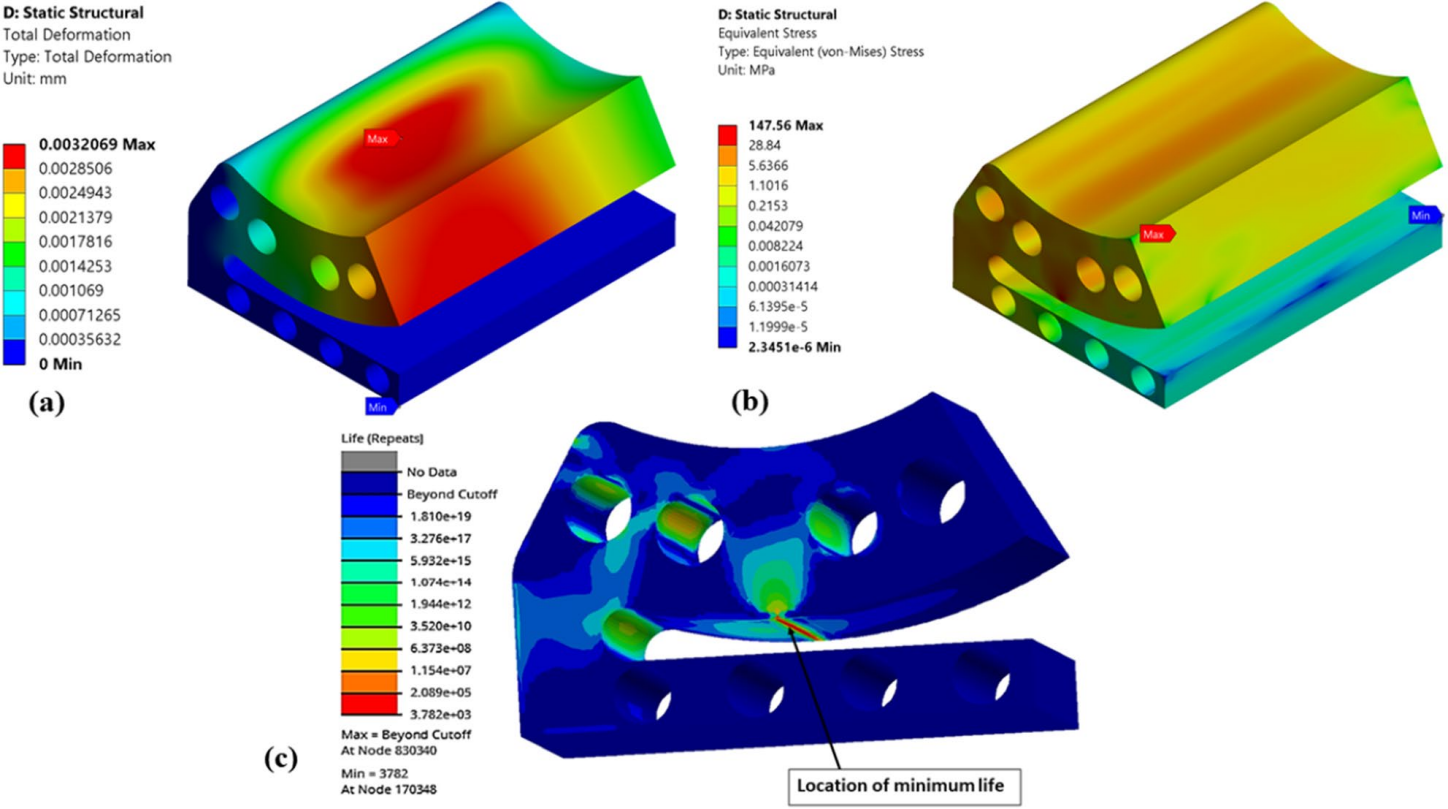
Numerical results of optimized (Type-5) single pad EAFF bearing (**a**) pad deformation, (**b**) von-Mises stress, and (**c**) fatigue life.

**Figure 19:**
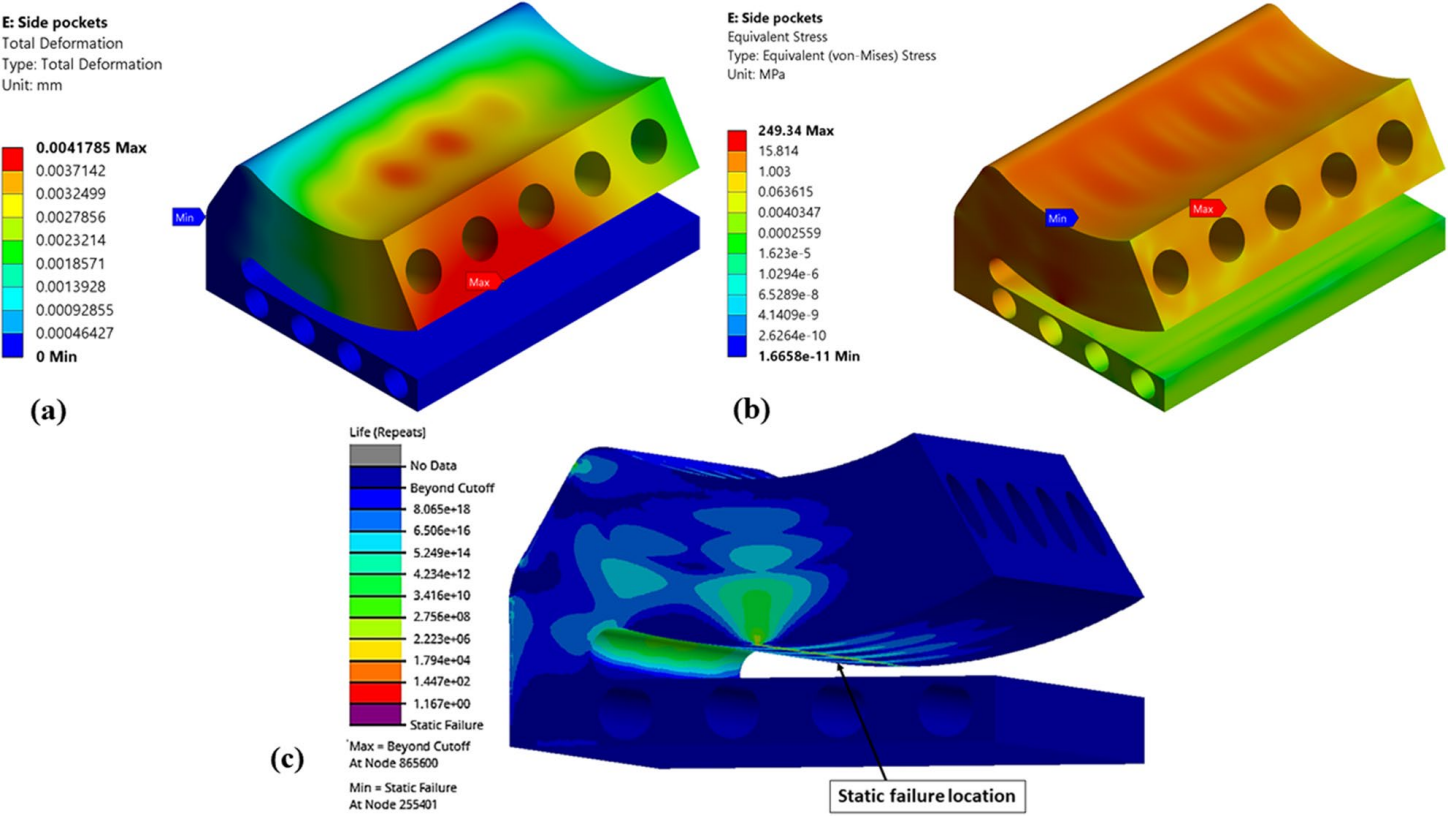
Numerical results of optimized (Type-6) single pad EAFF bearing (**a**) pad deformation, (**b**) von-Mises stress, and (**c**) fatigue life.

Figures 14, 15, 16, 17, 18 and 19 shows the numerical results of various pocket configurations (Type-1 to Type-6) used for the topology optimization followed by fatigue life prediction.

It is observed from Fig. 15 that for Type-2 pad geometry, the weight of the pad geometry is reduced by 38.14%, and stresses are increased by 62.4% compared to the original pad geometry. Even though stresses have increased in Type-2 pad geometry, on comparison of fatigue analysis results, it is found that there are no fatigue failures in the components. However, fatigue results showed that the line contact region is critical and might require attention. The rise in stresses at this region is mainly due to line contact between the special cam profile and the pad geometry, which can be reduced by geometrical modifications like fillet or rounding.

Further, shapes like elongated holes (Figs. 16 and 17) and circular holes (Figs. 18 and 19) have also been used to study the pad deformation, stresses, and fatigue life of the pad geometry. It is noted from the results (Fig. 19) that for Type-6 pad geometry, pad deformation and stresses developed are quite high, leading to static failure pad geometry. However, for other pad geometries with Type-3 and Type-4, the pad geometry is not undergoing failure. Hence, it is evident from the fatigue life prediction study that in comparison with other pad geometries, Type-2 pad geometry is recommended. However, Type-4 or Type-5 is better if we consider manufacturing aspects in terms of their suitability. In comparison to Type-4 and Type-5 pad geometries, Type-4 is better in terms of lower weight and stress. Hence, Type-4 (elongated holes) is recommended for better bearing performance with reduced bearing weight and can be easily manufactured using additive manufacturing techniques. The results of topology optimization and fatigue life predictions are summarized in Table 2.

**Table 2:** Results summary.

Pocket configurations	Mass (g)	Maximum deformation (mm)	Maximum stress (MPa)	Minimum life near the line contact region (cycles)
Non-optimized pad geometry (Type-0)	245.06	0.0036009	77.027	4.549e5
Type-1	177.63	0.004059	119.32	1.011e4
Type-2	151.57	0.0044036	125.09	8.013e3
Type-3	203.72	0.0029515	126.37	9.715e3
**Type-4**	**190.06**	**0.0036551**	**137.3**	**5.6913e3**
Type-5	193.08	0.0032069	147.56	3.782e3
Type-6	194.48	0.0041785	249.34	Static failure

Significant values are in bold.

## Conclusion

In this paper, to predict the fatigue life of a pad of an EAFF bearing, FSI and topology optimization technique based approach is adopted. Our investigation involves the fatigue analysis of various pad geometries using the two-way FSI results of a 60° pad of an EAFF bearing operating under high-speed conditions with an eccentricity ratio of 0.6. Initially, pad deformation and stresses developed for various pad geometry with pocket configurations are studied, and results are compared with non-optimized pad geometry. Various shapes of pockets (Type-1 to Type-6) are provided in the pad geometry to reduce the mass, and fatigue life is predicted using nCode fatigue analysis commercially available software. The following are the major findings of the current study.

- Based on the results of topology optimization (TO) technique, it is suggested that the weight of the pad geometry can be reduced by 42.91% compared to the original geometry. The TO technique recommends redesigning the pad geometry and, hence, incorporating pockets such as elongated holes and circular holes to achieve this weight reduction.
- The fatigue life of the non-optimized and optimized pads are predicted, and the results are compared. It is noted from the results that the presence of higher stresses is evident in the shorter fatigue life, making this area more susceptible to wear and the initiation of fatigue cracks.
- In the case of Type-2 pad geometry, there is a 38.14% reduction in the weight of the pad geometry, accompanied by a 62.4% increase in stresses when compared to the original pad geometry. Despite the elevated stresses in Type-2 pad geometry, a comparative analysis of fatigue results reveals no instances of fatigue failures in the components.
- In the case of Type-6 pad geometry, the pad experiences substantial deformation and high stresses, resulting in a static failure of the pad geometry. However, no failure was observed in the pad geometry for other pad configurations such as Type-3 and Type-4.
- Upon comparison among the various configurations evaluated, Type-4 pad geometry (four elongated holes in the axial direction of the shaft) performed better in terms of weight, stresses and fatigue life. Type-4 pad geometry reduces the weight by 22.45% compared to non-optimized pad weight. Also, it can be easily manufacturable using additive manufacturing techniques.

### Supplementary Information

Supplementary Figure S1.

## Data Availability

The datasets generated during and/or analyzed during the current study are available from the corresponding author upon reasonable request.
