# Supplementary figures and images for: Topological optimization and fatigue life prediction of a single pad externally adjustable fluid film bearing
Source: Sci Rep. 2024 Jun 10;14:13346. doi: 10.1038/s41598-024-64259-2 (PMC11164894; doi:10.1038/s41598-024-64259-2)

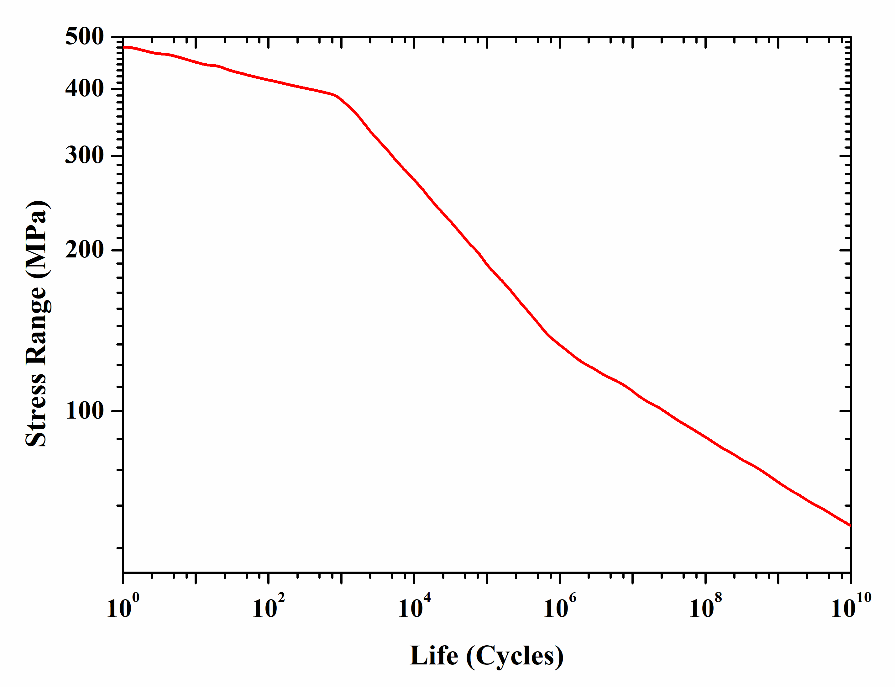


Figure S1: An S-N curve for bronze pad material

Supplement: Supplementary file 1 — Supplementary Figure S1. [file 41598_2024_64259_MOESM1_ESM.docx]
